# DAily time use, Physical Activity, quality of care and interpersonal relationships in patients with Schizophrenia spectrum disorders (DiAPASon): an Italian multicentre study

**DOI:** 10.1186/s12888-020-02588-y

**Published:** 2020-06-08

**Authors:** Giovanni de Girolamo, Matteo Rocchetti, Ilaria Maria Antonietta Benzi, Sara Agosta, Letizia Casiraghi, Clarissa Ferrari, Nicola De Franceschi, Ambra Macis, Silvia Pogliaghi, Fabrizio Starace

**Affiliations:** 1 Unit of Epidemiological and Evaluation Psychiatry, IRCCS Istituto Centro San Giovanni di Dio Fatebenefratelli, Brescia, Italy; 2 Department of Mental Health and Dependence, ASST of Pavia, Pavia, Italy; 3 Department of Brain and Behavioural Sciences, University of Pavia, Pavia, Italy; 4 Department of Mental Health and Dependence, AUSL of Modena, Modena, Italy; 5 Service of Statistics, IRCCS Istituto Centro San Giovanni di Dio Fatebenefratelli, Brescia, Italy; 6 Department of Information Engineering, University of Brescia, Brescia, Italy; 7 Department of Neurosciences, Biomedicine and Movement Sciences, University of Verona, Verona, Italy

**Keywords:** Schizophrenia, Daily time use, Experience sampling method

## Abstract

**Background:**

Schizophrenia spectrum disorders (SSD) are ranked among the leading causes of disabilities worldwide. Many people with SSD spend most of their daily time being inactive, and this is related to the severity of negative symptoms. Here, we present the 3-year DiAPAson project aimed at (1) evaluating the daily time use among patients with SSD living in Residential Facilities (RFs) compared to outpatients with SSD and to the general population (Study 1); (2) evaluating the quality of staff-patient relationships, its association with specific patient outcomes and the quality of care provided in RFs (Study 2); and (3) assessing daily activity patterns in residential patients, outpatients with SSD and healthy controls using real-time methodologies (Study 3).

**Methods:**

Study 1 will include 300 patients with SSD living in RFs and 300 outpatients; data obtained in these clinical populations will be compared with normative data obtained by the National Institute of Statistics (ISTAT) in the national survey on daily time use. Time use assessments will consist of daily diaries asking participants to retrospectively report time spent in different activities.

In Study 2, a series of questionnaires will be administered to 300 residential patients (recruited for Study 1) to evaluate the quality of care and staff-patient relationships, level of well-being and burnout of RFs’ staff, and quality of RFs using a European standardized questionnaire (QuIRC-SA).

In Study 3, the daily time use will be evaluated in a subgroup of 50 residential patients, 50 outpatients and 50 healthy controls using the Experience Sampling Method approach (participants will complete a brief questionnaire -about time use, mood and perceived energy- on a smartphone 8 times a day for 1 week) to compare retrospective and real-time reports. Moreover, their level of physical activity, sleep patterns, and energy expenditure will be monitored through a multi-sensor device.

**Discussion:**

This project is highly innovative because it combines different types of assessments (i.e., retrospective and real-time reports; multi-sensor monitoring) to trace an accurate picture of daily time use and levels of physical activity that will help identify the best therapeutic options promoting daily activities and physical exercise in patients with SSD.

**Trial registration:**

ISRCTN registry ID ISRCTN21141466.

## Background

Schizophrenia is one of the most disabling and severe mental disorders: while its incidence and prevalence are relatively low (McGrath and colleagues found a median prevalence of schizophrenia of 15.2 per 100,000 persons and a median lifetime morbid risk for schizophrenia of 7.2 per 1000 persons), its burden is substantial both for individuals and society [1, 2].

Here, we present the DiAPAson study, a 3-year-long project aimed at fostering our knowledge about the daily time use among people with Schizophrenia Spectrum Disorders (SSD) using different methodologies. The study will address the relationship between the daily time use and negative symptoms and will evaluate the level of Physical Activity (PA), which is known to be reduced in this clinical population. We will also investigate the quality of the staff-patient relationships and the quality of care in Residential Facilities (RFs), which provide long-term care in a country where all former mental hospitals have been shut down.

### Time and psychopathology: historical roots

The study of time and its perception among people with different types of mental disorders has long been an object of investigation for scientists and clinicians active in this field. The idea that temporality had a connection with the state of mind was initially formulated by Augustine of Hippo in his “Confessions” [3]. Later, over the nineteenth century, time became an essential variable for the emerging experimental or natural sciences until the philosopher Henri Bergson recognized the special status of the “subjective” time, in contrast to “objective” or chronological time: he called the former *“lived time”,* characterized by the expansion of vital momentum [4]. The prominent German philosopher Heidegger identified the very meaning of being in the future (as opposed to the past and the present) and thoroughly reflected on the temporality of human existence [5]. However, it was the French (born in Poland) psychiatrist Eugene Minkowski, relying on his Bergsonian readings, to work on the notion of time in psychiatry: in 1914, he published a monograph entitled *“The Essential Elements of Time-Quality*”*,* and in 1933, he produced his fundamental book entitled “Lived Time: Phenomenological and Psychopathological studies”, where he draws a vision of mental disorders based on temporality [6, 7]. Melancholy, in particular, was seen as a disorder where the past was frozen and no longer “passing”: in depression, time does not allow the present and the future to happen; on the contrary, in mania, time runs away, and the patient is unable to stop it. Overall, temporality in psychopathology can be considered the primary modality constituting the experience, that is, it gives shape to the experience, as well as to the symptoms of mental disorders.

### Daily time use in the general population and in people with severe mental disorders

“Daily time use” has been investigated since the late 1920s in the US, initially with a focus on economic productivity and inequalities in labour force and later expanded to assess the use of time among different socioeconomic groups [8]. Within a few years, this research area became of high relevance to describe social conditions and understand people’s behaviour, so that governments in different countries started to map how people used their time with “time use surveys” (TUS [9]). More recently, in 2000, Eurostat released a series of methodological guidelines for facilitating the data collection process and comparison over time of daily time use within and between countries (i.e., Harmonised European Time Use Surveys; HETUS).

Importantly, TUS validation has also allowed a comparison between normative and clinical samples. Several studies have shown that mental disorders are negatively linked to engagement in structured activities throughout the day [10, 11]. While it has long been known that many people with SSD are mainly inactive and apathetic, only recently the study of daily time use has started to be conducted with valid methodologies. These studies have shown that patients with SSD spend significantly less time engaging in structured, functional and social activities and more time resting and “doing nothing” than non-clinical populations [11, 12]. The disorder has a severe impact on social and leisure activities [13], producing a self-maintenance cycle for negative symptoms and influencing functional outcomes [14, 15]. All these findings highlight the importance of assessing daily time use, especially in at-risk populations such as individuals with SSD.

### How to assess daily time use in patients with schizophrenia?

Time-use surveys are usually conducted through semi-structured interviews where participants are asked to retrospectively report the time spent in a variety of daily activities. According to the latest HETUS guidelines, TUS provide comprehensive coverage of all activities in which the subject was involved during the day and facilitate epidemiological research into cross-national comparisons and trend analyses [8].

However, the use of clinical interviews and questionnaires for assessing time use in SSD patients might be biased due to their memory deficits [16, 17], with increased reporting of time engaged in activities while they are indeed “doing nothing” [12]. Thus, recent studies on time use in this clinical population have been based on prospective and real-time assessments, such as the Experience Sampling Method (ESM) approach [18]. The ESM approach provides an ecologically valid time sampling of self-reports that allows studying person-environment interactions in daily time use (e.g., places, people, activities, emotions). Furthermore, it gives instant estimates of current behaviour that are less vulnerable to recall biases [19–22]. This approach might be particularly efficient in evaluating SSD patients given their cognitive deficits and inclination to biases [23]. To assess instantaneous estimates of behaviour, current practice adds real-time measurements taken from accelerometers to ESM data [21, 24]. A recent study demonstrated the feasibility, sensitivity, reliability and validity of ESM methods to assess functioning in patients with SSD [18].

### Physical activity in people with SSD

SSD patients show higher morbidity and mortality compared with the general population [25]. Overall, these patients are at risk for physical comorbidities [26], mainly cardiovascular diseases, metabolic syndromes and diabetes, which cause a 7–20 year reduction in life expectancy [27–31]. Sedentary behaviour and low PA are risk factors for the development of these medical conditions and thus premature mortality [32, 33].

The effect of PA on chronic disease prevention is well documented [34]. In SSD patients, PA interventions have been shown to improve global cognition, working memory and attention [35] and increase white matter connectivity, brain volume, and serum brain-derived neurotrophic factor levels [36, 37]. Moreover, PA shows positive effects on psychological well-being and anxiety [38] and can improve mental and physical quality of life, reducing metabolic risk and overweight [39, 40]. Given that sedentary behaviour is an independent predictor (from PA) of cardiovascular diseases [41], emerging evidence suggests the importance of monitoring both PA and sedentary behaviour in SSD patients [42].

Collecting precise data on the actual levels of PA and sedentary behaviour is, therefore, of paramount importance in SSD patients. In this project, we will use objective measurement methods, such as (i) overall PA dose, assessed as the amount (minutes per day) and intensity (metabolic equivalents) and (ii) amount of sedentary time and sleep quantity and quality.

### Previous Italian studies investigating the time spent doing nothing among SSD patients

In Italy, some studies conducted among a large sample of patients staying in RFs have shown that most patients (in particular patients with SSD) spend little time in meaningful activities and have low levels of PA. In the nationwide PROGRES survey, de Girolamo et al. [43] found that 45% of the sample of 2932 residential patients was inactive, not even assisting with domestic activities in the facility.

Low levels of activity among RFs’ patients have been confirmed by a more recent longitudinal study (PERDOVE study, [44]) conducted among 403 patients hosted by 22 RFs in Northern Italy. This study evaluated in detail the daily time use, showing that (i) 30.8% of the sample spent most of their time alone and uninvolved in any activity; (ii) approximately 1/3 of the patients did “nothing” for more than 6 h/day; and (iii) negative symptoms in patients with SSD were highly prevalent. Similar findings have been obtained in other studies with patients living in RFs [45]. Surprisingly, there are very scant data about the amount of time spent in structured activities among outpatients with SSD, and this makes it impossible to understand whether the amount of daily time spent in meaningful activities is higher, comparable or lower among outpatients with SSD, living independently or with their families, compared to SSD patients living in RFs. Indeed, someone may argue that patients living in the community (often alone) may be more likely to be inactive than patients living in an assisted environment, where staff can stimulate patients and engage them in structured activities.

Given the importance of daily time use and its correlation with psychopathology, it is justified to state that time management represents one of the more informative prognostic indicators for patients with SSD.

### Quality of care and interpersonal relationships in residential facilities

For patients living in RFs, the quality of therapeutic relationships with staff plays a critical role and is a powerful predictor of patient outcomes [46–48]. Bordin [49] provided a useful framework to investigate the quality of the therapeutic relationship by introducing the concept of “working alliance”. The three main components of the therapeutic alliance are agreement on goals, assignment of tasks and development of bonds.

Another fundamental aspect to consider when exploring the quality of relationships in mental health settings is the level of burnout among staff members. An extensive literature has shown how working in mental health settings and experiencing and dealing with patients’ suffering on a daily basis places the clinical staff at risk for burnout [50, 51].

For all these reasons, it is essential to assess the overall quality of care and staff-patient relationships in long-term settings (such as RFs) and correlate these data to the overall functioning of patients living in these facilities.

## Hypotheses and objectives

The assumption underlying the DiAPAson project is that patients with SSD, living in RFs or living independently in the community, spend more time during the day “doing nothing” compared to the general population. We also assume that psychiatric severity, in particular, the severity of negative symptoms, will be positively correlated with the amount of time spent doing nothing.

Additionally, we hypothesize that significant discrepancies will be found comparing the assessment of daily time using a standard paper-and-pencil approach and the ESM methodology. Moreover, we assume that patients with SSD evaluated with real-time assessment through ESM and with appropriate body sensors (actigraphy) will show significant differences when compared to healthy controls: we suggest that specific patterns of associations related to the type of activity performed during the day, the perceived level of energy, PA level and mood will be found.

Finally, we assume that staff well-being and burnout levels will be associated with the quality of staff-patient relationships and specific patient outcomes and that ratings of RF’s quality using the QuIRC-SA will be positively associated with patients’ satisfaction.

### Objectives

The three main aims of the DiAPAson study, a 36-month-long project, are as follows:

#### Objective 1

To evaluate the daily time use (e.g., use of time spent in different activities) of patients with SSD living in RFs compared to outpatients with SSD and to a large, normative sample of the general population; to assess the relationship between the severity of psychiatric symptoms (in particular negative symptoms) and the amount of time spent doing nothing and being sedentary.

#### Objective 2

To assess the quality of staff-patient relationships for patients living in RFs and its relationship with the staff’s well-being and burnout, and to evaluate the quality of RFs rated by service managers using the Quality Indicator for Rehabilitative Care – Supported Accomodation (QuIRC-SA).

#### Objective 3

To compare real-time ESM data about daily activities with retrospective, paper-and-pencil assessments to evaluate the consistency between these two data collection methodologies, and to assess levels of PA with a multi-sensor monitor (actigraphy) in three groups (RF patients and outpatients with SSD compared to healthy subjects).

## Methods/Design

### Study design and management

Data collection will take place in many Departments of Mental Health (DMHs) and RFs across the country, and will start in June 2020; data collection will end on May 2021. The project will be coordinated by the IRCCS Fatebenefratelli of Brescia (coordinating centre), the DMH of Modena and the DMH of Pavia. To date, approximately over 35 DMHs and RFs have expressed written interest for inclusion in the project (see Acknowledgements for a list of participating facilities). Formal agreements are currently being finalized.

**Fig. 1:**
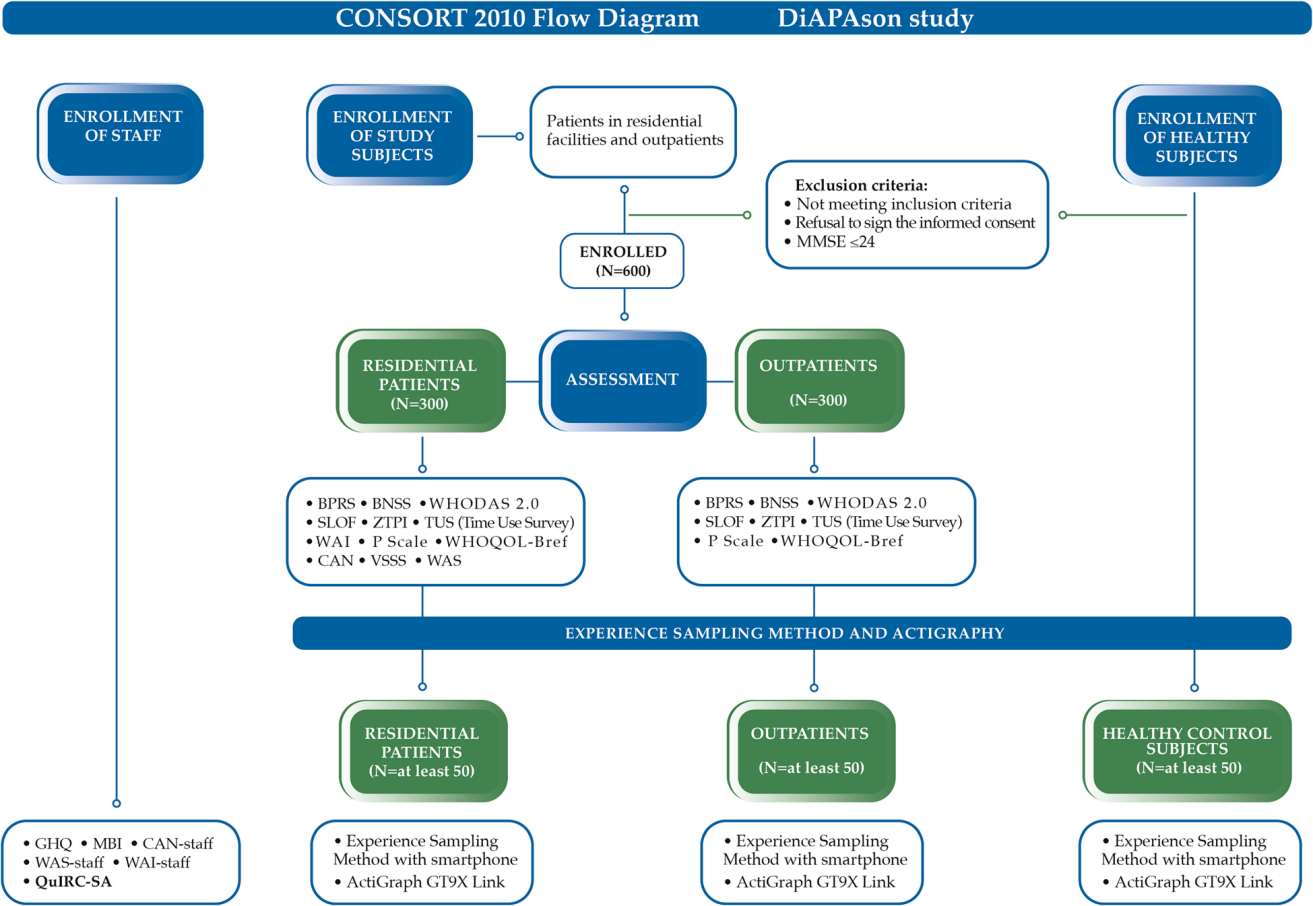
Flow-diagram showing procedures for enrolment and assessment of clinical subjects and healthy controls

Participants will be selected according to the inclusion and exclusion criteria shown in Table 1. They will approve and sign an informed consent form before inclusion in the study. The flow diagram shown in Fig. 1 provides a clear picture of enrolment and assessment procedures of all study subjects.

**Table 1:** Patients’ recruitment: inclusion and exclusion criteria

	Inclusion criteria	Exclusion criteria
All groups	- Age 20–55 years old; - Good knowledge of Italian language.	- Inability to provide informed consent (because of low education, or cognitive impairment); - Severe cognitive deficit (MMSE equal or lower than 24.0); - Lifetime diagnosis of substance use disorder according to DSM-5 criteria (APA, 2013 [52]); - History of clinically significant head injury; - Cerebrovascular, neurological disease.
G1 and G2	- SSD diagnosis according to DSM-5 (APA, 2013 [52]); - In charge to a RF or to a DMH as outpatient.

### Study 1 design

#### Participants

The sample will include 300 residential patients diagnosed with SSD (G1), matched by age and gender to 300 outpatients (G2). General population data will be extracted from the National Institute of Statistics (ISTAT) survey “*Uso del tempo quotidiano*”, a national survey that assesses how people spend their time, which was conducted in 2013 in Italy (ISTAT, https://www4.istat.it/it/prodotti/microdati#file_ricerca) in a representative sample of the Italian population including approximately 27,000 families (with a total of approximately 60,000 surveyed individuals) living in 508 municipalities (out of over 8000 Italian municipalities) of different sizes.

#### Measures and variables

A general assessment, together with demographics, will include measures of psychiatric symptoms (in particular negative symptoms), psychosocial functioning and time perception. Variables related to daily time use will include the number of daily hours spent in different activities: paid work, study, travel, doing nothing/resting, social/leisure activities, activities related to child/household care, voluntary activities, sport, and hobbies. Table 2 shows a comprehensive overview of the measures and areas of investigation; all reported instruments have been tested for validity and reliability in Italian: Table 2 shows the reference of the original paper for each instrument, and the reference of the Italian validation study. Time use assessments will consist of a pre-filled daily diary (to be completed twice a week, on a working day and on a weekend day) adapted from the ISTAT’s national survey on time use, asking participants to retrospectively report the daily time spent in different activities. The time spent in each activity will be calculated as number of hours per week.

**Table 2:** Assessment measures, domains and subjects receiving the assessment

Instrument/Reference (original and Italian validation)	Domain	Study group			
		Residential patients	Outpatients	Healthy controls	Staff
Mini Mental State Examination (MMSE) (Cockrell & Folstein, 2002 [53]; Measso et al., 1993 [54])	Cognitive functioning	X	X	X	
Brief Psychiatric Rating Scale (BPRS) (Overall and Gorham, 1962 [55]; Morosini et al., 1995 [56])	Psychiatric functioning	X	X		
Brief Negative Symptom Scale (BNSS) (Strauss et al. 2012 [57]; Mucci et al., 2015 [58])	Negative symptoms	X	X		
Specific Levels of Functioning Scale (SLOF) (Schneider et al., 1983 [59]; Mucci et al. 2014 [60])	Current functioning and behaviour	X	X		
World Health Organization Quality of Life Brief version (WHOQOL-Bref) (Whoqol Group, 1998 [61]; de Girolamo et al., 2000 [62])	Quality of life	X	X		
World Health Organization Disability Assessment Schedule 2.0 (WHODAS 2.0) (Gold, 2014 [63]; Federici et al., 2009 [64])	Psychosocial functioning	X	X		
Zimbardo Time Perspective (ZTPI-short form) (Zimbardo & Boyd, 2015 [65]; Laghi et al., 2009 [66])	Attitude towards time	X	X	X	
Positivity Scale (P Scale) (Caprara et al., 2012 [67])	Positivity assessment	X	X		
Time Use Survey (TUS) (see HETUS, 2019 [68])	Use of daily time	X	X	X	
Verona Service Satisfaction Scale (VSSS) (Ruggeri et al., 1993 [69])	Satisfaction with services	X			
Working Alliance Inventory Short Patient and Therapist versions (WAI-PT and WAI-TP) (Hatcher & Gillaspy, 2006 [70]; Lo Coco et al., 2011 [71])	Patients’ and staff relationships	X			X
Camberwell Assessment of Need Patient and Staff versions (CAN) (Phelan, 1995 [72]; Ruggeri et al., 1999 [73])	Health and social needs	X			X
Ward Atmosphere Scale (WAS) (Moos & Houts, 1968 [74]; Burti et al., 1990 [75])	Individual perception of the ward atmosphere	X			X
General Health Questionnaire (GHQ-12) (Goldberg, 1970 [76]; Fontanesi et al., 1985 [77])	General health evaluation				X
Maslach Burnout Inventory (MBI) (Maslach, 1981 [78]; Sirigatti et al., 1988 [79])	Level of Burnout				X
Quality Indicator for Rehabilitative Care (QuIRC-SA) (Killaspy, 2016 [80])	Quality of the rehabilitative service				X (RF’s manager)
Smartphone app	Use of daily time	X	X	X	
Smartphone app	Perceived energy	X	X	X	
Smartphone app	Mood	X	X	X	
Actigraphy	Physical activity	X	X	X	

#### Procedure

Clinicians and mental health workers at the participating sites will receive a training session on questionnaires’ administration. Participants will be asked to complete the instruments and will receive a 20-min training session on daily diary instructions. A web portal that will allow clinicians to directly fill in the study questionnaires will be developed, and this procedure will be guided with clear and simple user interfaces. The software will perform data validation to highlight possible errors. If there is no local access to an internet connection, it will be possible to use an off-line desktop application with the same functionalities but with the ability to locally save the collected data on a computer. Data will then be uploaded to the online database when the internet connection is available.

### Study 2 design

#### Participants

The sample will include 300 patients living in RFs (G1) and RF staff (approximately 1 staff member every 5 patients).

#### Measures and variables

Patients will be evaluated using a set of standardized instruments, as summarized in Table 2. The variables being studied will encompass quality of care and quality of relationships between patients and staff, level of well-being and level of burnout of RF staff, and quality of the facility assessed with the QuIRC-SA (i.e., living environment, treatments and interventions, therapeutic environment, self-management and autonomy, social interface, human rights, recovery-based practice).

#### Procedure

RF staff and RF managers will receive a specific training session about questionnaire administration. The facility manager will fill in the QuIRC-SA.

### Study 3 design

#### Participants

The sample will include a sub-group of at least 50 RF patients (drawn from G1), 50 outpatients (drawn from G2) and 50 healthy controls from the general population matched by age and gender. This latter group will also complete the TUS. Healthy controls will be recruited through ads, both using the project’s website and spreading the news of the study through social networks.

#### Measures and variables

We will perform a time use evaluation with the ESM data, and these reports will be compared with retrospective paper-and-pencil assessments to evaluate the degree of consistency between the two information sources. Patients and healthy controls performing the ESM assessment will also be asked to wear a multi-sensor device (actigraphy) to assess levels of PA over the course of 1 week. For the ESM assessment, participants will be equipped with smartphones where a DiAPAson app will be installed. The answer to the smartphone’s prompt will allow easy evaluation of the following:

- the specific activity being carried out by the patient (e.g., paid work, leisure, resting/doing nothing);
- mood (i.e., rating of different adjectives on a scale from 0 to 100; e.g., “sad”, “happy”, etc.);
- perceived level of energy (i.e., rating of different adjectives on a scale from 0 to 100; e.g., “active”, “tired”, etc.).

#### Procedure

Each participant will receive a 30-min training session on the ESM procedure. Participants will install the DiAPAson app on their smartphones (or will be lent a smartphone if they do not have one) and will be asked to carry the device with them over a 7-day period. The smartphone will generate a prompting auditory “recall” signal, sending a notification 9 times a day at random intervals between 7 AM and 11 PM. Participants will have to complete the ESM questionnaire on the smartphone screen (it takes approximately 1 min and has to be completed no later than 30 min after being prompted). ESM reports will allow the assessment of some of the variables examined in the TUS, as well as perceived energy and mood.

After a short training on PA assessment, participants will be asked to wear a multi-sensor device, ActiGraph GT9X Link (manufactured by ActiGraph, Pensacola, FL 3250, USA), continuously over 7 consecutive 24-h periods. The device will be worn on the non-dominant arm and set to record 60-s epochs, computing a total activity count for each active minute of the day. This method relies on a wearable device (combining accelerometer with measurements of heat production and skin conductivity) that measures physiological variables and bodily movements over prolonged periods of time. An algorithm converts raw data into estimates of Energy Expenditure (EE), which are expressed both in kcal/min and Metabolic EquivalenTs (METs) [81]. The multi-sensor device has been shown to provide accurate results for estimating EE and time spent in various activities and to provide valid estimates of EE in the general population [82]. The following PA parameters will be measured: (i) average physical activity: the average of all active minutes during days and nights; (ii) total idle time as well as sleeping (including sleep latency, sleep efficiency and total sleep time): average number of inactive minutes between morning alarm and time when going to bed; (iii) energy expenditure; (iv) MET rates; and (v) body position. These data will be collected as integrated activities every 60 s using arbitrary units.

Measurements via smartphone and actigraphy will be simultaneous (i.e., during the same week). Outpatients and healthy controls participating in the ESM and PA monitoring will receive a € 25 reimbursement in supermarket vouchers as compensation for travel expenses to receive the training. All these data will be saved in a secure database for future analysis. At the end of the assessment week, the project web portal will be used to upload the armband data.

### Recruitment and data collection

Eligible participants will be recruited from a large number of RFs across the entire country, allowing meaningful comparisons of facilities and services located in different areas. Consent forms will be collected according to the local ethical committee requirements. Participants will be matched by age, gender and diagnosis. Both residential patients and outpatients will be recruited by treating clinicians; outpatients meeting inclusion criteria will be consecutively invited to join the study. Similar number of participants will be recruited at each participating centre, also accounting for the specific characteristics of each participating facility. Overall, patients to be recruited in each center will range from 15 (smaller ones) to 40 subjects (bigger ones).

As spelled out in the original protocol approved by the funding agency, data collection will last 8 months. Questionnaires will be administered as participants are enrolled. Patients and controls participating in Study 3 (and Objective 3) will be invited to start the ESM and the monitoring of PA immediately after questionnaire administration. The administration of tools to be filled by clinicians for all patients (BPRS, BNSS and SLOF) will last approximately 1 hour; the administration of tools to be filled by clinicians only for residential patients (e.g., WAI-TP, CAN and WAS) will last approximately one additional hour. Self-administered tools (e.g., WHOQOL-Bref, WHODAS 2.0, ZTPI, P Scale, CAN, VSSS, WAI-PT, WAS) for patients will take approximately 15 min each, and can also be filled at different times. Training for both TUS, ESM and actigraphy administration will last approximately 40 min and will be organized in small groups of 2–3 participants.

### Data analyses

#### Study 1

Data on daily time use in the different groups will be compared using parametric and non-parametric methods depending on the distribution of the investigated variables. Correlation estimates (Pearson or Spearman) will be used to assess the associations between the severity of psychiatric symptoms and the amount of sedentary time.

Sample size was calculated based on Cella and colleagues’ study [12], which compared patients with SSD (*N* = 170) and a general population sample (*N* = 1124). Their results showed that the average time spent “doing nothing” (target) in the patient group was 5.17 h per day, while for the general population, it was 0.76 h with a standard deviation of 5 for both groups. With such data, the effect size for a linear model is 0.44. As a precaution, assuming a lower effect size equal to approximately 0.15, using ANCOVA (including age and gender in the model) with three groups, and choosing a power of 0.8, the estimated total sample size is equal to *N* = 435. Moreover, considering a total of approximately 25 different recruitment centres, the correction for the design effect (DE = 1 + (435/25–1)*0.02 = 1.3, with an intraclass correlation coefficient equal to 0.02) leads to a sample size of about *N* = 570, which was increased to *N* = 600 (300 for each group) to account for possible dropouts of about 10%. Finally, 300 matched subjects randomly sampled from the ISTAT general population sample will be added as third group, for a total of *N* = 900 subjects evaluated for daily time use.

#### Study 2

To evaluate how the level of burnout of the staff affects the quality of the therapeutic relationships with the staff, correlation estimates (Pearson or Spearman) will be used. The influence of the burnout level (categorized as high/low) of staff (each staff member associated with a patient) on the quality of the therapeutic relationship with patients (WAI-pt) will be investigated by comparing groups (patients and staff in RFs) with generalized linear or linear models. To evaluate the associations between the quality of RFs rated by service managers and patient experiences of care, in accordance with Killaspy and colleagues [83], we will perform multilevel modelling analyses of associations between service user ratings (level 1 data) as dependent variables with unit QuIRC-SA domain ratings (level 2 data) as independent variables. The models will also include potential mediators (i.e., age, gender, and level of severity of psychiatric symptoms as assessed in Study 1).

#### Study 3

Appropriate tests will be used to compare the daily time use with routine methods and with ESM according to the distribution of the data. In particular, frequencies and corresponding chi-square tests, as well as Cohen’s kappa concordance index, will be used to compare ESM reports and retrospective assessments and to assess the degrees of consistency between the two sources of information.

The sub-sample chosen for Study 3 (*N* = 150, including 50 randomly chosen from Group 1, 50 randomly chosen from Group 2 and 50 healthy subjects) is in line with the sample size of Merikangas and colleagues [84]. Multilevel models (MLM [85]) will be used to simultaneously evaluate within- and between-subject associations, taking into account repeated measurements. In addition, cross-lagged analyses will be carried out to examine the directionality of the measured effects. Comparisons of PA quantity variables between groups will be evaluated using linear and generalized linear models.

## Discussion

The overarching focus of the DiAPAson project is to collect valid and reliable research data to suggest recommendations for improving mental health services in Italy. In particular, previous studies, everyday clinical experience, and more importantly, the perspectives of people with SSD highlight the urgency of formulating innovative intervention strategies.

Currently, research on people with SSD in the field of long-term care is lagging behind more “fashionable” areas of research in psychiatry and neuroscience. This project, in line with previous health services research conducted in Italy [43, 44], aims to shed light on some crucial clinical dimensions of SSD that have substantial translational implications. The study includes several highly innovative components and combines various types of evaluation to draw an accurate picture of daily time use and PA levels in a heterogeneous clinical population. In Italy, only a single study, with a small sample (*N* = 27), has been conducted with ESM on patients with severe mental disorders [86]. For this reason, the present study represents a critical innovation for mental health research. Second, no studies have ever been conducted in Italy using multi-sensors for PA in patients with SSD (or any other mental disorders), and our study methodology represents a novelty. Finally, the large sample size (*N* = 600) may overcome the main limitations of previous studies on daily time use.

### Strengths

#### An in-depth overview of the daily time use in patients with SSD in RFs and outpatients

The DiAPAson project will offer a unique opportunity for an in-depth examination of the daily time use in different subgroups of people with SSD compared with healthy controls. The project will also clarify whether there is a relationship between the level of functioning of these patients and their daily time use. Moreover, it will critically and objectively assess the effect of the everyday life “setting” for patients with SSD (RFs vs community). Overall, data gathered from the DiAPAson project might be very relevant if we consider that time management is one of the more informative outcomes for patients with SSD.

#### Real-time exploration of daily time use, PA and mood

To the best of our knowledge, DiAPAson is the first study investigating daily time use in patients with SSD using both a retrospective assessment (diaries and TUS) and real-time momentary assessment (ESM and actigraphy). Importantly, in SSD patients, studies conducted to date have shown that even when negative symptoms are relevant, patients may be more active than it is generally thought [87, 88]. For these reasons, a real-time survey will allow us to explore multiple levels of complexity at once. Moreover, ESM guarantees the possibility of capturing individual variability (within-subject) for several types of data and not only a global overview (between-subject).

Passive monitoring of PA is another essential aspect of the project. To our knowledge, this is the first study investigating these critical aspects using ESM together with actigraphy in this specific clinical population. The only study using a similar approach was previously conducted with patients suffering from bipolar disorder [84].

#### A multiple-perspective approach to mental health and care services

One of the crucial aspects that the DiAPAson project focuses on is the complexity of the interactions that shape daily life in RFs. Moreover, to assess the quality of staff-patient relationships in patients living in RFs, it is essential to acknowledge not only multiple aspects of the residents but also to shed light on the staff’s general well-being. It has long been known that a staff member who is burned out and unsatisfied with work is likely to face many difficulties in dealing with patients suffering from severe, long-term mental disorders.

### Limitations

The evaluation process includes many tools and will take some time to be completed, especially for the participants who will be enrolled in all three studies. Although this may lead to high refusal rates, previous experience with health services research carried out in Italy has shown that, due to the high degree of collaboration between staff and patients, refusal rates can be kept very low [43, 44, 89, 90].

The facilities participating in the study will not be randomly chosen for obvious organizational reasons and therefore might not be representative of all Italian mental health services. During data analysis, appropriate corrections will be introduced to limit possible biases.

### Study status

The current protocol is updated to February 2020. Recruitment will take place from June 2020 to July 2021.

## Data Availability

The datasets generated and/or analysed during the current study will be available in the UniData – Bicocca Data Archive repository (https://www.unidata.unimib.it/?lang=en).

## Abbreviations

DMH: Department of Mental Health
DMHD: Department of Mental Health and Dependence
DSM-5: Diagnostic and Statistical Manual of Mental Disorders 5th Edition
EE: Energy expenditure
ESM: Experience sampling method
G1: Group 1
G2: Group 2
HETUS: Harmonised European Time Use Surveys
ISTAT: Italian National Institute of Statistics
PA: Physical activity
RF: Residential facility
SMD: Severe mental disorders
SSD: Severe Mental Disorder
TUS: Time Use Survey
